# 

**Published:** 1911-03

**Authors:** 


### CONTENTS.

ORIGINAL COMMUNICATIONS—

Local Anaesthesia. By Dr. W. L. Cooke, Columbus, Ga. .......................... 607

Vaccine Therapy with Special Reference to So-Called Surgical Tuberculosis. By Lewis M. Gaines, A. B., M. D., Atlanta, Ga. .............................. 615

What Are the Indications at the Present Time for the Direct Tranfusion of Blood? E. G. Jones, M. D. and D. F. Winn, M. D., Atlanta, Ga. ........ 621

Varicocee in the Male. (Part 2. By Aime Paul Heineck, M. D., Chicago, Ill. .. 626

Salvarsan, or "606," Ehrlich's New Remedy for Syphilis, By Edgar M. Ballenger, M. D., Atlanta, Ga. .................................................. 641

EDITORIALS .................................................................... 654

BOOK REVIEWS ................................................................ 656

